# Physical Literacy for Communities (PL4C): physical literacy, physical activity and associations with wellbeing

**DOI:** 10.1186/s12889-023-16050-7

**Published:** 2023-06-29

**Authors:** Yiling Tang, Beatrix Algurén, Chelsea Pelletier, Patti-Jean Naylor, Guy Faulkner

**Affiliations:** 1 School of Kinesiology, Faculty of Education, University of British Columbia, Vancouver, BC Canada; 2 Department of Food and Nutrition, and Sport Science, Faculty of Education, University of Gothenburg, Göteborg, Sweden; 3 School of Health Sciences, University of Northern British Columbia, Prince George, BC Canada; 4 School of Exercise Science, Physical and Health Education, University of Victoria, Victoria, BC Canada

**Keywords:** Physical literacy, Children, Physical activity, Mental health, Movement behavior, PLAY tools, Movement skills, Self-efficacy, Mediation

## Abstract

**Background:**

Physical literacy (PL) is considered an important determinant of children's physical activity through which health benefits may be derived. The purpose of this study is to describe a sample of Canadian children’s baseline levels of PL and movement behaviors, and explore whether the associations between PL and their mental wellbeing, if any, are mediated by moderate-to-vigorous physical activity (MVPA).

**Methods:**

All grade two children in 14 elementary schools in the West Vancouver School District, Canada were invited to participate in a two-year longitudinal project. PL was assessed through PLAYfun and PLAYself tools. Physical activity was measured by wrist-worn accelerometers (GT3X + BT) for seven days. Children's mental well-being was assessed using the Strengths and Difficulties Questionnaire (SDQ). A score of total difficulties was aggregated for internalizing and externalizing problems.

**Results:**

A total of 355 children aged 7–9 (183 boys, 166 girls, 6 non-binary) participated with 258 children providing valid accelerometer data. Children exhibited an average of 111.1 min of MVPA per day, with 97.3% meeting the physical activity guidelines. Approximately 43% (108/250) of participants were meeting the Canadian 24-h movement guidelines. Children were at an ‘emerging’ level of overall physical competence (45.8 ± 5.6) and reported a mean score of 68.9 (*SD* = 12.3) for self-perceived PL, with no significant differences between boys and girls. PL was significantly associated with MVPA (*r* = .27) and all SDQ variables (*r*s = -.26—.13) except for externalizing problems. Mediation analyses showed PL was negatively associated with internalizing problems and total difficulties when the association with MVPA was considered. However, the mediating role of MVPA was found only between PL and internalizing problems, β = -.06, 95%CI [-.12, -.01].

**Conclusions:**

Although most of our sample was physically active and showed higher adherence to 24-H movement guidelines than comparable population data, the motor competence and self-perceived PL of our sample were similar to those of previous studies. PL has an independent association with children’s internalizing problems and total difficulties. Ongoing assessment will investigate the relationships between PL and children’s mental health from a longitudinal perspective.

**Supplementary Information:**

The online version contains supplementary material available at 10.1186/s12889-023-16050-7.

## Introduction

The independent benefits of physical activity and limited sedentary behavior on children's physical and mental health have been widely acknowledged [1, 2]. Regular physical activity participation is beneficial for enhancing muscular strength and bone health [3, 4], improving cardiovascular fitness [5], and preventing multiple chronic diseases such as cancer [6], obesity [7, 8], and type II diabetes [8]. In addition, physical activity appears to be effective for reducing depression/depressive symptoms and improving physical self-perceptions [9, 10], while also providing opportunities for children to develop their social skills and improve cognitive performance (i.e., concentration) and academic achievement [11–14]. Research suggests sedentary behavior also has an independent association with increasing health problems [15, 16]. Sedentary behavior is defined as any behavior in a sitting, reclining, or lying posture that requires an energy expenditure of ≤ 1.5 metabolic equivalent of tasks (METs) during waking hours [17]. The Canadian 24-H Movement Guidelines for Children and Youth recommend children accumulate at least 60 min of moderate-to-vigorous physical activity (MVPA) per day and limit their screen-based sedentary behavior to two hours each day [18]. These guidelines highlight the importance of considering all movement behaviors as a whole and suggest children aged 5–13 should get 9 to 11 h of good-quality sleep each night. Meeting all three behavior guidelines is associated with improved overall health as opposed to meeting none or part of the recommendations [19]. However, a recent systematic review and meta-analysis found that a fifth of young people across 23 countries do not meet any of the recommendations and only 10.3% of children meet the recommended 24-H movement guidelines for all three behaviors worldwide [20]. In Canada, only 2.6% of Canadian children aged 5–17 were meeting all three recommendations since the outbreak of COVID-19 [21].

Physical literacy (PL) is a multi-dimensional concept that has gained increasing attention in the field of public health as it is theoretically identified as an important determinant of physical activity across the lifespan [22, 23]. Many different conceptual and operational definitions of PL have been adopted in the literature [24, 25]. Of these definitions, PL is most frequently defined as “the motivation, confidence, physical competence, knowledge and understanding to value and take responsibility for engagement in physical activities for life”. This definition was adopted by the International Physical Literacy Association [23, 26]. PL consists of four domains: affective, cognitive, physical, and behavioral, all of which are interrelated [27]. It is considered as the capability for a physically active lifestyle that can be developed at any age [27, 28]. PL is particularly important in early childhood, a critical period for the development of foundational movement skills [29] and the establishment of physical activity habits [30]. Physically literate individuals participate in higher levels of physical activity, spend more time in sports, and have lower levels of sedentary behavior [31, 32]. Therefore, PL can have a positive impact on children’s overall health and on public health [24]. As illustrated in a conceptual model linking PL, physical activity and health, the effect of PL on health is hypothesized to be fully mediated by physical activity [33]. A cross-sectional study by Caldwell et al. [32] demonstrated that positive associations between PL assessed by the Physical Literacy Assessment for Youth (PLAY) tools (e.g., PLAYfun and PLAYself), and health indicators (e.g., health-related quality of life) were partially mediated by MVPA. Less is known as to whether PL offers direct benefits on children’s mental health.

Emerging evidence highlights the importance of promoting mental health in early childhood [34]. Globally, around 10–20% of children and adolescents are affected by mental health problems, which contribute significantly to the global burden of disease [34]. Mental health problems in early childhood can have a negative impact on a child’s development and can lead to social and emotional difficulties [35–37]. Prevention of mental health problems in childhood offers a compelling opportunity to impact overall health across the life span and alleviate the public health burden of mental disorders [38]. A study by Blain, Curran [39] demonstrated a positive relationship between PL assessed by a validated measure, the Canadian Assessment of Physical Literacy (CAPL), and psychological well-being in adolescents (mean age = 12.84 years), while there is only one study evaluating the relationship between PL, children’s (mean age = 10.31 years) psychosocial well-being, and the mediating role of MVPA [40]. Melby, Nielsen [40] found that PL was significantly associated with all aspects of psychosocial well-being, whereas the associations were not mediated by MVPA. More research is warranted in examining the relationship between PL, physical activity, and mental health among children.

An opportunity to examine these relationships is available in the context of the Physical Literacy for Communities (PL4C) initiative in Canada (https://physicalliteracy.ca/communities). The aim of the PL4C initiative is to support the development of PL in children and youth ages 2–18 years across selected Canadian communities. PL4C also aims to establish multi-sector community partnership tables and build capacity to support delivery of community PL programs in community settings (e.g., community programs and schools). A key component of the capacity building is providing training and resources to sport, recreation, education and health program leaders. One established community is embedded within the West Vancouver School District, which consists of 14 public elementary and three secondary schools in British Columbia, Canada. This district adopted a school-based PL initiative which included two School District embedded mentors who provide ongoing support for elementary school teachers in the district. In a longitudinal dose response design, the PL of grade two students in all 14 West Vancouver elementary schools is being assessed at three time points over two years. School implementation levels of support by the mentors is being tracked at the same time to explore the relationship between changes in PL and in-school support by the mentors. Embedded within the larger national PL4C initiative, this more focused evaluation (referred to as ‘WAVES PL’) provides a pragmatic opportunity to explore the co-variation of PL, physical activity and mental health in the sample over a two-year period.

**Fig. 1:**
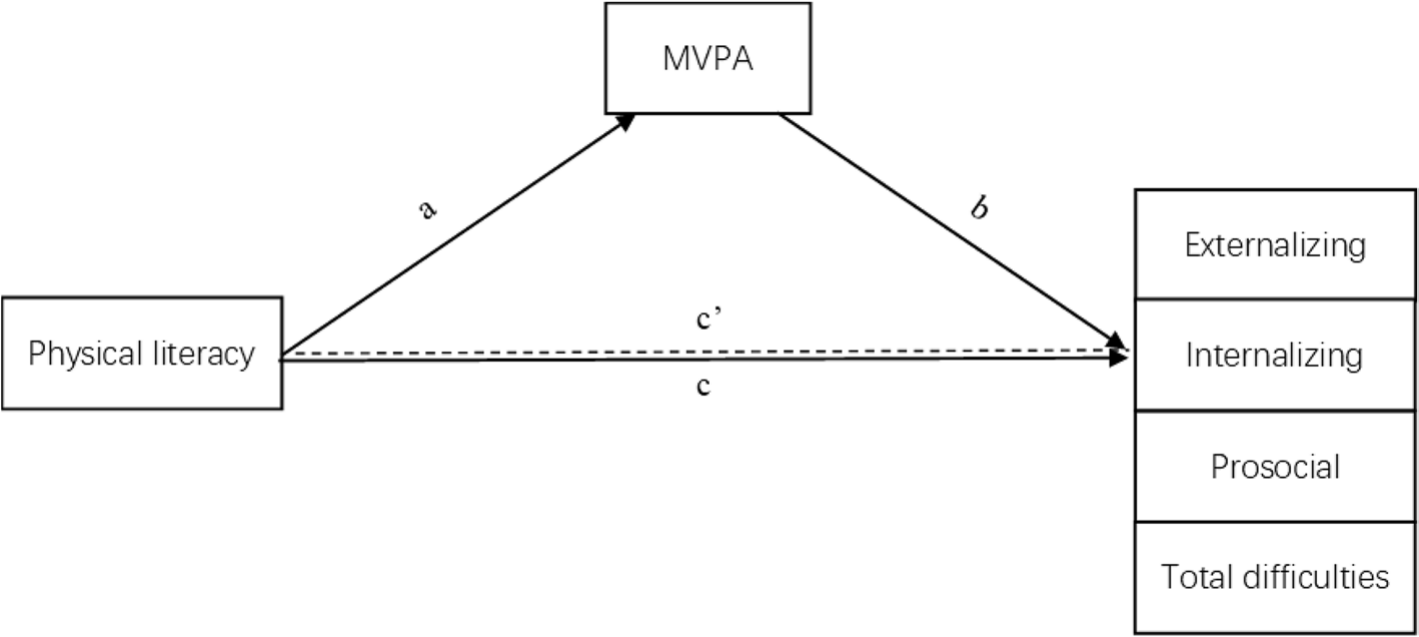
Hypothesized mediation model between PL, MVPA, and mental health

The objectives of this study are to 1) describe the WAVES PL sample at baseline with a focus on PL and movement behavior outcomes, and 2) to cross-sectionally assess associations between children’s PL, device-measured physical activity, and mental health. Regarding our second research objective, we hypothesized that associations between PL and mental health would be partially mediated by MVPA (Fig. 1).

## Methods

### Study design and recruitment

All 14 elementary schools in the district were invited and consented to participate in this project. Three data collection time points across two years were scheduled (Spring 2022, Spring 2023, and Spring 2024). Data in the current study were collected as baseline data (April 2022 – May 2022). All children in Grade 2 or Grade 2/3 split classrooms and their parents (*n* = 473) were invited to participate. Written consent forms and assent forms were provided by parents and children respectively. School visits were scheduled at teachers’ convenience to minimize interference with class routines. All children who completed in-school PL assessments and provided valid accelerometry data were provided with a $20 gift card as compensation. A donation for physical activity resources was also provided to each school. The study received approval from the appropriate Institutional Research Ethics and School Boards.

### Measures and data collection

#### Child-level measurements

##### Physical literacy

Physical literacy was assessed using the Physical Literacy Assessment for Youth (PLAY) tools specifically developed for children aged 7 or older [41]. PLAY consists of six different tools (PLAYfun, PLAYbasic, PLAYself, PLAYparent, PLAYcoach and PLAYinventory) comprising workbooks, forms and tracking sheets that were designed for assessing PL. For the present longitudinal project, PLAYself and PLAYfun were applied.

*PLAYself*. The PLAYself questionnaire is used to explore children's perceptions of their PL indicating self-efficacy for physical activity participation [42]. PLAYself consists of 22 questions that fall within four subsections: (1) environment, where children are asked to rank their ability to do sports and activities in different physical environments on a 5-point Likert scale ranging from “never tried” (1) to “excellent” (5); (2) statements about doing sports and activities based on cognitive and affective factors, where the children are asked to rank how well they agree on a 4-point Likert scale ranging from “not true at all” (1) to “very true” (4); (3) children’s ranking of importance of literacies (i.e., literacy, numeracy, and physical literacy) in school, at home with family, and with friends each on a 4-point Likert scale ranging from “strongly disagree” (1) to “strongly agree” (4); and (4) children’s perceived fitness level with “disagree (1)/agree (2)” response categories to a single item, if it restricts their participation in activities they have chosen. For each of the first three subsections a score can be calculated as well as a total score for PLAYself. The total PLAYself score is the average across the scores of each subsection excluding the fitness question. The higher the score (0—100), the higher the self-perceived PL. The PLAYself tool has shown excellent test–retest reliability (ICC = 0.81–0.84), good–excellent internal consistency (Person Separation Index = 0.70–0.82), and good convergent validity (correlation range: 0.42–0.78) among 300 Canadian children aged 8–14 years [43]. Researcher facilitation and rephrasing/clarification were used to guide completion of the PLAYself tool for the present study.

*PLAYfun*. The PLAYfun tool assesses children's individual performance of 18 fundamental movement tasks across five domains, i.e., a) running (3 tasks), b) locomotor (5 tasks), c) object control—upper body (4 tasks), d) object control – lower body (2 tasks), and e) balance, stability, and body control (4 tasks) [44]. The performance quality of each task was assessed using continuous criterion-referenced visual analogue scales divided into four categories, ranging from initial (< 25), emerging (25—< 50), competent (50—< 75), to proficient (75—100). Typically, a score above 50 indicates an entry-level ("acquired") level of motor competence, whereas a lower score indicates a "developing" level of motor competence. For each of the five skill domains, an average score of the domain-specific tasks can be calculated, as well as the total PLAY*fun* score that is the average score across all five domains. The PLAYfun tool has been shown to be a valid and reliable measurement tool for measuring PL in Canadian children and youth aged 6–14 [45–47]. The inter-rater reliability of the PLAYfun tool is very good to excellent (ICC = 0.87–0.94) [45–47] and has shown good internal consistency (α = 0.87) with two raters [47]. Prior to use of the tool for this study, all research assistants completed training sessions. Each task was assessed independently by two research assistants and the final task score was calculated as the average of the two assessors’ scoring. Five out of the 18 tasks (one from each domain) that constitute the short-form of PLAYfun (known as PLAYbasic) were provided through the semiannual assessments by two PL specialists. The inter-rater reliability for each skill between two PL mentors was found to be very good (ICC = 0.81–0.89), and acceptable to very good (ICC = 0.72–0.87) for the two research assistants in the present study, as examined by two-way random effects ICC.

##### Physical activity

Actigraph GT3X + BT (ActiGraph, Pensacola, FL), a triaxial accelerometer, was placed on participants’ non-dominant wrist for better compliance [48–50]. Participants were instructed to wear the accelerometer for consecutive 7 days except for water-based activities (e.g., swimming, showering, and bathing). The GT3X + BT was initialized to record raw accelerations at a 30 Hz sampling rate. Chandler’s cut points [51] based on vector magnitude were used to classify different intensities of physical activity and sedentary behavior. The cut points were developed for children aged 8–12 using non-dominant wrist-worn accelerometers and were recommended by Migueles, Cadenas-Sanchez [52]. Physical activity was categorized into: ≤ 305 (sedentary), 306 – 817 (LPA; light physical activity), 818 – 1968 (MPA; moderate physical activity), ≥ 1969 (VPA; vigorous physical activity). The same epoch length (5-s) used when developing Chandler’s cut points [51] was selected as epoch length can influence activity counts [52].

#### Parent-reported measurements

Participants' parents were asked to complete a questionnaire, either on paper or online. The parent questionnaire consists of questions related to demographic information (e.g., parents’ and their partner’s (if applicable) age, work conditions, education, and cultural background). Questions also asked for the quantity of time their child spent in sports and time spent outdoors, screen time during weekends and school days, usual mode of travel to and from school, and sleep duration. Two scales, the Activity Support Scale for Multiple Groups (ACTS-MG) and the Neighborhood Environment Walkability Scale for Youth (NEWS-Y), were included as potential factors impacting opportunities for PL development. This information was beyond the scope of the present study and will be presented elsewhere.

Finally, children’s mental health was parent-reported using the Strengths and Difficulties Questionnaire (SDQ) [53]. The SDQ consists of 25 items that fall within five hypothesized subscales, namely a) hyperactivity/inattention, b) conduct problems, c) emotional symptoms, d) peer relationship problems, and e) prosocial behavior. The first four subscales represent negative aspects of children’s psychosocial well-being, and the prosocial subscale presents the positive aspect. Participants were asked to respond on a 3-point Likert scale ranging from “Not true” (0) to “Certainly true” (2). Five out of the 25 items were reversed coded, i.e. “Not true” (2) to “Certainly true” (0). Scores of the five scales are the sum of subscale-specific five items ranging from 0 to 10, where a higher score implies more problems for negative aspects and less problems for the positive prosocial aspect.

### Data reduction and treatment

Along with the PLAYself assessments, participants’ age and gender were collected. Participants were categorized into three gender groups: boys, girls, and non-binary. Four participants completed parent surveys but provided the consent and assent forms after the scheduled school visits, and therefore, were not involved in the PLAYself, PLAYfun, and physical activity assessments. In addition, 14 participants never received an accelerometer as they were absent during the school visits. One participant lost the accelerometer. Accelerometer data of 336 participants were downloaded and processed on ActiLife v. 6.13.4 (ActiGraph, Pensacola, FL). Consistent with most previous studies [54], a minimum of four valid days (at least three school days and one weekend day) with at least 10 h of wear time per day during waking hours were required to be valid accelerometer data. The Cole-Kripke sleep algorithms were employed for determining waking hours [55]. Any time periods with 30 consecutive minutes or more of zero counts were considered non-wear time, with no interruption allowed [56]. A total of 78 participants did not meet the wear time criteria and were excluded from the subsequent physical activity analyses. Wear time, steps, minutes spent in different intensities of physical activity per day (e.g., LPA (min/d)), and the percentage of each intensity of physical activity (e.g., LPA (%)) were calculated. Following the Canadian 24-H Movement Guidelines for Children and Youth [18], participants were categorized into yes/no (1/0) regarding a) meeting physical activity guidelines (PAG), defined as an average of 60 min of MVPA per day measured by accelerometer; b) meeting the screen time guidelines (SCG), defined as 2 h or less of daily screen time on average reported by parents; c) meeting the sleep guidelines (SG) defined as 9–11 h of sleep per day reported by parents; and d) meeting the Canadian 24-H Movement Guidelines when yes in a, b, and c above. As suggested [18, 57], participants who met two other operational PAG definitions that considered accumulating 60 min of MVPA on all days (e.g., 4/4 and 5/5) and at least 6 out of 7 days were also presented.

For the parent-reported measurements, answers about parents’ work condition were categorized into three groups: full-time work (at least one parent works full-time), part-time work (at least one parent works part-time, but no parent works full-time), and out of work (no parent works neither full-time nor part-time). Parent’s highest education represents the highest education level between parent and their partner (if applicable).

In addition, although the SDQ has been widely used for evaluating children mental health worldwide [58], less consensus has been reached on the internal structure of the SDQ. Comparing the original five-factor structure, an alternative internalizing/externalizing/prosocial model appears to be more appropriate for the current study with a low-risk young sample. Internalizing score (range 0 – 20) and the externalizing score (range 0–20) were calculated by summing the emotional and peer problem scales, and the conduct and hyperactivity scales respectively. The fifth subscale, prosocial, was used individually. This three-factor model demonstrated good convergent and better discriminant validity than five subscales in low-risk children aged 5–16 [59]. In addition, a total difficulties score was used, which has been found to be valid in measuring overall mental health of British children aged 5–16, as well as children of different ages from various countries around the world [58–60]. The total difficulties score (range 0—40) was calculated by adding the scores of the first four scales except the prosocial behavior scale where 15–17 indicates borderline and 17 and above indicates abnormal. A score of 0 – 14 was labeled as low (normal) level of total difficulties.

Finally, in light of the multidimensional definition of PL, it may be more appropriate to incorporate different PLAY tools to determine children's overall PL rather than using PLAY tools individually [24]. Therefore, for our second research objective, adapted from Caldwell et al. (2020), a PL composite score was calculated as the sum of PLAYfun and PLAYself z-scores [32].

### Statistical analyses

All statistical analyses were processed using SPSS ver. 28 (IBM SPSS, Chicago, IL). Descriptive statistics were expressed as mean and standard deviation for continuous variables and proportions for categorical variables. The Chi-square tests were used for testing independence of categorical variables by gender or whether participants met or did not meet 24-H movement guidelines. Normality was assessed for continuous variables using Shapiro–Wilk tests, kurtosis, skewness, and Q-Q plots. Independent sample t-tests were used for comparing means of normal-distributed variables. The Mann–Whitney U tests were used for non-normal distributed variables. The sample size of non-binary participants was very small (*n* = 6) and differed from the other two groups (183 boys and 166 girls). Thus, this group was excluded from gender-dependent analyses (e.g., Chi-square tests, t-tests, and Mann–Whitney U tests). One-way repeated measures ANOVA tests were conducted to investigate the differences of movement behaviors between school days and weekends among boys or girls, or stratified by meeting the 24-H movement guidelines. Friedman's tests, a non-parametric equivalent to one-way repeated measures ANOVA, were used to determine whether non-normal distributed variables (i.e., MVPA (min/d), MVPA (%), screen time during school days and weekends) were different for boys and girls and for children who met or did not meet 24-H movement guidelines.

Mediation analyses were conducted using the Hayes PROCESS macro in SPSS ver.28 to determine whether MVPA mediates the association between composite PL score and different aspects of mental health. Pearson’s correlation and Spearman’s correlation were first used to investigate relationships between parametric variables and non-parametric variables respectively. The composite PL, MVPA, and mental health indicators were entered as independent variables (X), mediators (M), and dependent variables (Y) respectively. Internalizing, externalizing, prosocial, and total difficulty were examined independently. Gender and age were included as covariates since previous literature demonstrated that boys engage in more MVPA than girls and physical activity decreases with age, though these associations were not significant in our study (Tables 2 and 5). Both unstandardized effects (B) and standardized effects (β) were demonstrated. All *p*-values < 0.05 were considered statistically significant. Bootstrapping was set to 10,000 samples. The bootstrapped 95% confidence interval (CI) was used to determine the indirect effect.

## Results

### Overview

Table 1 presents participants’ and their parents’ demographics. A total of 355 participants (183 boys, 166 girls, 6 non-binary) and their parents completed consent and assent forms. This reflects a response rate of 75% of eligible families. The average age of the children was 7.5 years (*SD* = 0.5, range: 7 – 9 years), most of them had at least one sibling (*n* = 199, 58.7%). Six children (1.7%) reported experiencing long-lasting disabilities including: autism (*n* = 3), attention deficit hyperactivity disorder (*n* = 1), poor vision (*n* = 1), and development coordination disorder (*n* = 1). Most parents (*n* = 253, 74%) and their partners (*n* = 191, 59%) were aged 30 to 44, working either full-time (62.9%) or part-time (34.8%), with mainly a university (48.5%) or graduate-level education (45.0%). Most parents self-identified as Asian (46.0%), followed by European (29.0%), from another cultural background (20.6%), or Indigenous (0.6%).

**Table 1:** Participants and their parents’ demographics

**Characteristic**	**N**	**Overall**^**a**^**, *N* = 355**	**Boy**^**a**^**, *N* = 183**	**Girl**^**a**^**, *N* = 166**	**Statistical test**^**b**^	**Non-binary**^**a**^**, *N* = 6**
**Age (year)**	355				*t*(347) = 1.47, *p* = .07	
Mean (SD)		7.5 (0.5)	7.5 (0.6)	7.4 (0.5)		7.7 (0.5)
**Disability**	343				Χ^2^ (1, *N* = 337) = 0.55, *p* = .46	
Yes		6 (1.7%)	4 (2.3%)	2 (1.2%)		0 (0.0%)
No		337 (98.3%)	170 (97.7%)	161 (98.8%)		6 (100.0%)
**Siblings**	339				Χ^2^ (3, *N* = 333) = 1.54, *p* = .67	
0		64 (18.9%)	36 (20.9%)	27 (16.8%)		1 (16.7%)
1		199 (58.7%)	98 (57.0%)	98 (60.9%)		3 (50.0%)
2		60 (17.7%)	29 (16.9%)	30 (18.6%)		1 (16.7%)
3		16 (4.7%)	9 (5.2%)	6 (3.7%)		1 (16.7%)
**Parent's age (year)**	342				Χ^2^ (2, *N* = 336) = 0.06, *p* = .97	
Under 30		2 (0.6%)	1 (0.6%)	1 (0.6%)		0 (0.0%)
30 to 44		253 (74.0%)	128 (73.6%)	121 (74.7%)		4 (66.7%)
45 or over		87 (25.4%)	45 (25.9%)	40 (24.7%)		2 (33.3%)
**Partner's age (year)**	324				Χ^2^ (2, *N* = 318) = 0.03, *p* = .99	
Under 30		4 (1.2%)	2 (1.2%)	2 (1.3%)		0 (0.0%)
30 to 44		191 (59.0%)	97 (59.9%)	92 (59.0%)		2 (33.3%)
45 or over		129 (39.8%)	63 (38.9%)	62 (39.7%)		4 (66.7%)
**Parent's highest education**	340				Χ^2^ (3, *N* = 334) = 0.20, *p* = .98	
Elementary		0 (0.0%)	0 (0.0%)	0 (0.0%)		0 (0.0%)
Secondary School		8 (2.4%)	4 (2.3%)	4 (2.5%)		0 (0.0%)
College		14 (4.1%)	8 (4.7%)	6 (3.7%)		0 (0.0%)
University		165 (48.5%)	84 (48.8%)	79 (48.8%)		2 (33.3%)
Graduate School		153 (45.0%)	76 (44.2%)	73 (45.1%)		4 (66.7%)
**Parent's work conditions**	310				Χ^2^ (2, *N* = 306) = 2.13, *p* = .35	
Full-time		195 (62.9%)	103 (66.0%)	88 (58.7%)		4 (100.0%)
Part-time		108 (34.8%)	49 (31.4%)	59 (39.3%)		0 (0.0%)
Out of work		7 (2.3%)	4 (2.6%)	3 (2.0%)		0 (0.0%)
**Parent's culture background**	335				Χ^2^ (5, *N* = 329) = 3.17, *p* = .67	
Indigenous		2 (0.6%)	2 (1.2%)	0 (0.0%)		0 (0.0%)
Hispanic		10 (3.0%)	6 (3.5%)	4 (2.5%)		0 (0.0%)
Asian		154 (46.0%)	81 (47.4%)	71 (44.9%)		2 (33.3%)
European		97 (29.0%)	49 (28.7%)	47 (29.7%)		1 (16.7%)
African		3 (0.9%)	2 (1.2%)	1 (0.6%)		0 (0.0%)
Other		69 (20.6%)	31 (18.1%)	35 (22.2%)		3 (50.0%)

^a^n / N (%); Mean (SD)

^b^Chi-square test; Mann–Whitney tests and independent sample t-tests between groups

### Movement behaviors

Overall, 258 (76.8%; 128 boys, 126 girls, 4 non-binaries) met the wear time criteria. Table 2 shows participants’ movement behaviors levels. On average, participants wore the accelerometer for 800.0 min/day (*SD* = 57.5). A total of 251 participants (97.3%; 125 boys, 122 girls, 4 non-binaries) accumulated an average of at least 60 min of MVPA, and were considered meeting the PAG. Of them, 150 children (58.1%, 77 boys, 73 girls) accumulated at least 60 min of MVPA every day. In addition, a total of 90 participants provided valid PA data daily across 7 days (data not shown). Of them, 44 (48.9%, 23 boys, 21 girls) accumulated at least 60 min of MVPA every day, and 76 (84.4%, 33 boys, 43 girls) were physically active on at least 6 out of 7 days. On average, children spent 457.8 min/d, 2.1 h/d, and 9.8 h/d on total sedentary behavior, recreational screen use, and sleep respectively. Most children [90.4% (311/344)] met the SG and 48.5% (165/340) met the SCG based on daily screen time. Overall, 43.2% (108/250) of children met all three 24-H movement guidelines.

**Table 2:** Participants’ movement behavior levels

**Characteristic**	**N**	**Overall, *N* = 355**^**a**^	**Boy, *N* = 183**^**a**^	**Girl, *N* = 166**^**a**^	**Statistical test**^**b**^	**Non-binary, *N* = 6**^**a**^
Accelerometer wear time (min/d)	258	800.0 (57.5)	804.9 (60.2)	793.9 (54.3)	*t*(252) = 1.53, *p* = .13	838.5 (47.9)
Steps	258	13,984 (2269)	14,603 (2272)	13,321 (2108)	***t*(252) = 4.66, *p* < .001**	15,069 (1047)
Sedentary (min/d)	258	457.8 (57.0)	461.0 (57.1)	454.4 (57.3)	*t*(252) = 0.92, *p* = .36	458.4 (50.4)
Sedentary (%)	258	57.2 (6.0)	57.3 (5.7)	57.3 (6.4)	*t*(252) = 0.01, *p* = .99	54.7 (5.4)
LPA (min/d)	258	231.2 (31.9)	231.2 (30.9)	230.2 (32.3)	*t*(252) = 0.25, *p* = .80	264.9 (42.2)
LPA (%)	258	28.9 (3.5)	28.8 (3.6)	29.0 (3.4)	*t*(252) = -0.50, *p* = .62	31.6 (4.6)
MPA (min/d)	258	97.9 (25.5)	97.6 (25.6)	98.0 (25.9)	*t*(252) = -.13, *p* = .89	103.4 (16.1)
MPA (%)	258	12.2 (2.9)	12.1 (2.9)	12.3 (3.0)	*t*(252) = -.65, *p* = .52	12.3 (1.5)
VPA (min/d)	258	13.2 (6.7)	15.1 (7.3)	11.3 (5.5)	***t*(236) = 4.72, *p* < .001**	11.8 (1.8)
VPA (%)	258	1.6 (0.8)	1.9 (0.9)	1.4 (0.7)	***t*(240) = 4.59, *p* < .001**	1.4 (0.2)
MVPA (min/d)	258	111.1 (30.3)	112.7 (30.9)	109.3 (30.1)	*t*(252) = 0.89, p = .37	115.2 (16.4)
MVPA (%)	258	13.9 (3.5)	14.0 (3.5)	13.7 (3.6)	*t*(252) = 0.47, *p* = .64	13.7 (1.5)
LMVPA (min/d)	258	342.3 (55.3)	343.9 (52.6)	339.5 (57.8)	*t*(252) = 0.64, *p* = .53	380.1 (53.8)
Sleep duration (h/d)	344	9.8 (0.9)	9.7 (0.8)	9.8 (0.9)	*U* = 13,223.00, *p* = .23	9.6 (0.5)
Daily screen time (h/d)	340	2.1 (1.3)	2.3 (1.3)	2.0 (1.2)	***U* = 11,360.00, *p* = .003**	2.7 (1.1)
Meeting PAG	258	251 / 258 (97.3%)	125 / 128 (97.7%)	122 / 126 (96.8%)	Χ^2^ (1, *N* = 254) = 0.16, *p* = .69	4 / 4 (100%)
Meeting SG	344	311 / 344 (90.4%)	158 / 175 (90.3%)	147 / 163 (90.2%)	Χ^2^ (1, *N* = 338) = 0.001, *p* = .98	6 / 6 (100%)
Meeting SCG	340	165 / 340 (48.5%)	76 / 172 (44.2%)	87 / 162 (53.7%)	Χ^2^ (1, *N* = 334) = 3.03, *p* = .08	2 / 4 (33.3%)
Meeting 24-H movement behavior guidelines	250	108 / 250 (43.2%)	48 / 122 (39.3%)	59 / 124 (47.6%)	Χ^2^ (1, *N* = 246) = 1.70 *p* = .19	1 / 4 (25.0%)

*Min/d*   minutes per day, *h/d* hours per day, *LPA* Light physical activity, *MPA* Moderate physical activity, *VPA* Vigorous physical activity, *MVPA* Moderate-to-vigorous physical activity, *PAG* Physical activity guidelines, *SG* Sleep guidelines, *SCG* Screen time guidelines

^a^n / N (%); Mean (SD)

^b^Chi-square test; Mann–Whitney tests and independent sample t-tests between groups

Independent t-tests showed boys accumulated more average daily steps, VPA (min/d), and VPA (%) than girls (*p*s < 0.001). A Mann–Whitney U test indicated that boys (*Md* = 2.0, *n* = 172) spent more time on.screens than girls (*Md* = 1.8, *n* = 162), *p* = 0.003. There were no significant differences between boys and girls in MVPA (min/d), MVPA (%), and amount of sleep (*p*s = 0.23—0.64).

In supplementary file 1, comparisons between boys and girls, as well as between children who met and did not meet the 24-H movement guidelines in their movement behaviors during school days and weekends are presented. Additionally, participants' movement behaviors were compared between school days and weekends, stratified by gender, as well as their achievement of 24-H movement guidelines (Supplementary 2). In particular, the non-parametric Friedman test indicated that boys and girls accumulated more minutes of MVPA during school days (boys: *M* = 117.7, *SD* = 30.3; girls: *M* = 113.1, *SD* = 31.8) than weekends (boys: *M* = 101.5, *SD* = 45.4; girls: *M* = 99.9, *SD* = 36.7). Similar differences were observed when grouping participants by meeting 24-H movement guidelines (*p*s < 0.001). Overall, regardless of whether boys or girls did or did not meet the 24-H movement guidelines, they were more sedentary (including spending more time on screens) and less physically active over the weekend than during the week (*p*s ≤ 0.001—0.03).

### Physical literacy

Table 3 presents descriptive statistics for the PLAYself, PLAYfun, and the composite PL variables, as well as comparisons by gender. On average, children’s motor competence levels for all five PLAYfun domains were in the ‘late emerging phase’ from the lowest, locomotor (*M* = 41.9, *SD* = 8.2), to the highest, lower body object control domain (*M* = 48.0, *SD* = 6.6). Boys scored higher in upper body and lower body object control domains than girls (*p*s < 0.001). In contrast, boys displayed lower locomotor scores than girls, t(312) = -2.76, *p* = 0.01. There were no significant differences in children’s overall motor competence (PLAYfun total score) and the other two domains (Running and Balance) between boys and girls (*p* = 0.11 -0.97). Children reported a mean score of 68.9 for self-perceived PL. The mean values of PLAYself subscales ranged from PL self-description (*M* = 67.6, *SD* = 15.6) to environmental participation (*M* = 71.5, *SD* = 15.3) over the entire sample. There were no differences in any PLAYself components and PLAYself total score between boys and girls (*p* = 0.62—0.82). There was no significant difference between boys (*M* = 0.08, *SD* = 1.59) and girls (*M* = -0.06, *SD* = 1.39) in the composite PL score, t(311) = 0.80, *p* = 0.43.

**Table 3:** PLAYfun, PLAYself, and physical literacy

		**Overall**^**a**^	**Boys**^**a**^	**Girls**^**a**^	**Statistical test**^b^	**Non-binary**^**a**^
**PLAYfun variables**	N	320	167	147		6
Running		45.6 (5.8)	45.6 (5.8)	45.5 (5.9)	*t*(312) = 0.04, *p* = .97	46.0 (4.8)
Locomotor		41.9 (8.2)	40.8 (7.7)	43.3 (8.4)	*t*(312) = -2.76, *p* = .01*	39.9 (11.3)
Upper Body		45.5 (7.7)	47.9 (7.8)	42.9 (6.7)	*t*(312) = 6.11, *p* < .001***	45.6 (8.8)
Lower Body		48.0 (6.6)	49.4 (6.5)	46.5 (6.3)	*t*(312) = 3.94, *p* < .001***	47.8 (6.7)
Balance		47.9 (7.2)	47.8 (7.7)	48.1 (6.4)	*t*(312) = -0.44, *p* = .66	47.1 (10.2)
**PLAYfun**		45.8 (5.6)	46.3 (5.6)	45.3 (5.3)	*t*(312) = 1.62, *p* = .11	45.3 (7.7)
**PLAYself variables**	N	334	175	153		6
Environment		71.5 (15.3)	71.0 (15.7)	72.0 (14.9)	*t*(326) = -0.59, *p* = .56	76.4 (12.8)
Self-Description		67.6 (15.6)	67.4 (16.4)	67.8 (14.7)	*t*(326) = -0.18, *p* = .86	70.4 (17.1)
Rank of Literacy		68.7 (16.4)	68.3 (17.8)	69.2 (14.8)	*t*(326) = -0.50, *p* = .62	68.6 (18.0)
Fitness					Χ^2^ (1, *N* = 328) = 1.06, *p* = .30	
Disagree		67 (20.1%)	32 (18.3%)	35 (22.9%)		0 (0.0%)
Agree		267 (79.9%)	143 (81.7%)	118 (77.1%)		6 (100.0%)
**PLAYself**		68.9 (12.3)	68.5 (13.5)	69.2 (10.9)	*t*(324) = -0.49, *p* = .62	71.1 (11.1)
**PL Composite Score**		.02 (1.50)	.08 (1.59)	-.06 (1.39)	*t*(311) = 0.80, *p* = .43	.09 (1.98)

*PL* Physical literacy

^*^p < .05, **p < .01, ***p < .001

^a^n / N (%); Mean (SD)

^b^Chi-square test; Independent sample t-tests between groups

### Mental health

Table 4 shows descriptive statistics of the SDQ variables by gender or the attainment of 24-H movement guidelines. A significantly higher hyperactivity score was found in boys (*Md* = 3.0, *n* = 173) than girls (*Md* = 2.0, *n* = 163), *p* < 0.001. A similarly significant difference was found in aggregated externalizing score between boys and girls, *p* = 0.004. There were no significant differences in emotional and peer problems scores individually, as well as the aggregated internalizing problems between boys and girls (*p*s = 0.66—0.89). Overall, children experienced a low level of psychological difficulties, with boys scoring higher than girls, *p* = 0.02. In addition, a higher prosocial score was found for girls (*Md* = 9.0, *n* = 163) compared to boys (*Md* = 9.0, *n* = 173), *p* = 0.03. Compared to participants who met the 24-H Movement guidelines (*Md* = 2.0, *n* = 107; *Md* = 3.0, *n* = 107), children who did not meet all three recommendations had significantly higher scores for hyperactivity (*Md* = 3.0, *n* = 141) and aggregated externalizing problems (*Md* = 4.0, *n* = 141), *p*s = 0.003—0.004.

**Table 4:** Strength and Difficulties variables by gender and 24-H movement behavior guidelines

	**Gender**					**Meeting 24-H movement guidelines**		
	**Overall**^a^	**Boys**^a^	**Girls**^a^	**Statistical test**^**b**^	**Non-binary**^a^	**Yes**^a^	**No**^a^	**Statistical test**^b^
**N**	342	173	163		6	107	141	
Emotional Problems	1.5 (1.7)	1.6 (1.8)	1.5 (1.7)	*U* = 13,723.50, *p* = .66	1.5 (1.8)	1.4 (1.6)	1.5 (1.7)	*U* = 7460.00, *p* = .88
Conduct Problems	1.3 (1.5)	1.4 (1.6)	1.2 (1.2)	*U* = 13,402.00, *p* = .42	2.5 (2.3)	1.1 (1.4)	1.3 (1.4)	*U* = 6763.50, *p* = .15
Hyperactivity	3.3 (2.3)	3.7 (2.4)	2.8 (2.1)	*U* = 11,136.00, *p* < .001***	3.2 (2.9)	2.6 (2.0)	3.5 (2.4)	*U* = 5966.50, *p* = .004**
Peer Problems	1.4 (1.5)	1.4 (1.6)	1.3 (1.4)	*U* = 13,949.50, *p* = .86	2.0 (1.8)	1.4 (1.5)	1.1 (1.4)	*U* = 6655.00, *p* = .10
Prosocial	8.3 (1.8)	8.1 (1.8)	8.5 (1.8)	*U* = 12,239.50, *p* = .03*	9.0 (0.9)	8.4 (1.8)	8.3 (1.7)	*U* = 7291.50, *p* = .64
Total difficulties	7.5 (4.8)	8.1 (5.2)	6.8 (4.3)	*U* = 12,020.00, *p* = .02*	9.2 (7.2)	6.5 (4.6)	7.4 (4.5)	*U* = 6515.50, *p* = .07
Externalizing	4.6 (3.2)	5.1 (3.4)	4.0 (2.8)	*U* = 11,582.00, *p* = .004**	5.7 (4.5)	3.7 (2.9)	4.8 (3.2)	*U* = 5914.50, *p* = .003**
Internalizing	2.9 (2.6)	2.9 (2.8)	2.8 (2.5)	*U* = 13,975.00, *p* = .89	3.5 (3.2)	2.8 (2.5)	2.6 (2.5)	*U* = 7165.50, *p* = .49

“Yes” means that participants met the Canadian 24-H movement guidelines; “No” means that participants did not meet any or all three 24-H movement guidelines

^*^p < .05, **p < .01, ***p < .001

^a^Mean (SD)

^b^Mann-Whitney tests

### Associations between main variables

The associations between key variables are presented in Table 5. PLAYfun and PLAYself were positively correlated, *r*(317) = 0.15, *p* = 0.01. PLAYfun, PLAYself, and the composite PL score were significantly associated with MVPA (min/d) and MVPA (%) but not sedentary behavior (min/d). The percentage of time spent in sedentary behavior showed a positive association with both PLAYfun and composite PL scores. In addition, MVPA (min/d) and MVPA (%) were negatively associated with internalizing problems, *r*(248) = -0.22, *p* < 0.001 and *r*(248) = -0.20, *p* = 0.002, respectively, but not with other mental health indicators. Sedentary time (%) was negatively correlated with externalizing scores, *r*(248) = -0.13, *p* = 0.04, but was positively correlated with internalizing scores,* r*(248) = 0.15, *p* = 0.02.

**Table 5:** Correlations among variables

		1	2	3	4	5	6	7	8	9	10	11	12
1	Age	–											
2	MVPA (min/d)	-.03	–										
3	MVPA (%)	-.04	.96**	–									
4	Sedentary (min/d)	.04	-.47**	-.66**	–								
5	Sedentary (%)	.20	-.81**	-.86**	.82**	–							
6	Prosocial^a^	.11*	.03	.06	-.08	-.04	–						
7	Externalizing^a^	-.08	.09	.08	-.10	-.13*	-.36**	–					
8	Internalizing^a^	-.02	-.22**	-.20**	.03	.15*	-.22**	.29**	–				
9	Total difficulties^a^	-.07	-.06	-.05	-.06	-.01	-.37**	.85**	.72**	–			
10	PLAYself	.04	.15*	.15*	-.06	-.09	.10	.09	-.11	.002	–		
11	PLAYfun	.14*	.30**	.26**	-.01	-.16*	.10	-.07	-.28**	-.19**	.15**	–	
12	PL composite score	.13*	.27**	.25**	-.04	-.14*	.13*	.01	-.26**	-.13*	.76**	.76**	–

*Min/d* minutes per day, *MVPA* Moderate-to-vigorous physical activity, *PL* Physical literacy

^*^p < .05 level, 2-tailed; **p < .01 level, 2-tailed

^a^Spearman’s correlations

No associations were found between PLAYself and any mental health indicators individually (*p*s = 0.06—0.97). PLAYfun was significantly negatively associated with internalizing problems (*r*(305) = -0.28, *p* < 0.001) and total difficulties (*r*(305) = -0.19, *p* < 0.001), but not prosocial (*r*(305) = 0.10, *p* = 0.08) and externalizing problems (*r*(305) = -0.07, *p* = 0.19). In addition, PL composite score was significantly associated with all SDQ variables except for externalizing. There was a positive association between PL and the prosocial score, *r*(304) = 0.13, *p* = 0.03. In addition, higher PL was correlated with less internalizing problems (*r*(304) = -0.26, *p* < 0.001) and lower total difficulties (*r*(304) = -0.13, *p* = 0.02).

### Mediation analyses

**Fig. 2:**
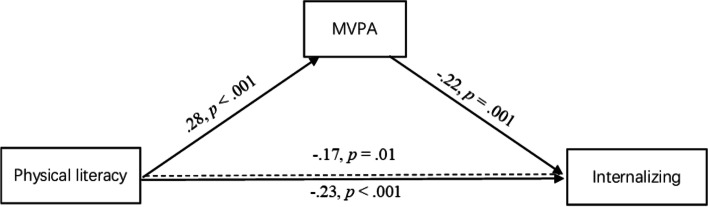
MVPA mediates the associations between PL and internalizing problems

Results indicated that PL was only significantly and negatively associated with internalizing problems and total difficulties (See Table 6). PL was a significant predictor of internalizing problems, B = -0.39, SE = 0.11, 95%CI[-0.61, -0.18], β = -0.23, *p* < 0.001, and the relationship was still significant but attenuated after controlling for MVPA (mediator), B = -0.29, SE = 0.11, 95%CI[-0.51, -0.08], β = -0.17, *p* = 0.01. MVPA was negatively associated with internalizing problems, B = -0.02, SE = 0.01, 95%CI[-0.03, -0.01], β = -0.22, *p* = 0.001. The results of the indirect effect based on 10,000 bootstrapping samples showed a significant negative relationship between PL and internalizing problems mediated by MVPA, B = -0.10, β = -0.06, standardized bootstrapped 95%CI[-0.12, -0.01], indicating partial mediation (Fig. 2). An increase of one standard deviation in PL was associated with a decrease of 0.06 standard deviations on internalizing problems through the indirect effect of MVPA.

**Table 6:** Mediation analyses

	Outcome: Externalizing						Outcome: Internalizing					
Paths	**β**	**B**	**SE**	**t**	***p***	**95% CI**	**β**	**B**	**SE**	**t**	***p***	**95%CI**
PL—> MVPA (a)	.28	5.57	1.27	4.39	** < .001**	3.07, 8.07	.28	5.57	1.27	4.39	** < .001**	3.07, 8.07
PL—> outcome (c’)	-.06	-.12	.14	-.87	.39	-.40, .15	-.17	-.29	.11	-2.65	**.01**	-.51, -.08
MVPA—> outcome (b)	.10	.01	.01	1.54	.12	-.003, .02	-.22	-.02	.01	-3.33	**.001**	-.03, -.01
Total effect (c)	-.03	-.06	.14	-.46	.65	-1.37, .13	-.23	-.39	.11	-3.63	** < .001**	-.61, -.18
Direct effect (c’)	-.06	-.12	.14	-.87	.39	-.40, .15	-.17	-.29	.11	-2.65	**.01**	-.51, -.08
	**β**	**B**	**BootSE**	**Boot 95%CI**		**Standardized Boot 95%CI**	**β**	**B**	**BootSE**	**Boot 95%CI**		**Standardized Boot 95%CI**
Indirect effect (a*b)	.03	.06	.04	-.01	.15	-.01, .07	-.06	-.10	.05	-.21	-.02	-.12, -.01
**Model summary**	**R**^**2**^	**MSE**	**F**	**p**			**R**^**2**^	**MSE**	**F**	**p**		
Outcome	.03	9.57	1.76	.14			.10	5.99	6.47	** < .001**		
Total effect model	.02	9.63	1.54	.21			.06	6.25	4.73	**.003**		
	Outcome: Total difficulties						Outcome: Prosocial					
**Paths**	**β**	**B**	**SE**	**t**	**p**	**95% CI**	**β**	**B**	**SE**	**t**	**p**	**95%CI**
PL—> MVPA (a)	.28	5.57	1.27	4.39	** < .001**	3.07, 8.07	.28	5.57	1.27	4.39	** < .001**	3.07, 8.07
PL—> outcome (c’)	-.14	-.41	.21	-2.02	**.04**	-.82, -.01	.11	.12	.08	1.58	.12	-.03, .28
MVPA—> outcome (b)	-.05	-.01	.01	-.75	.46	-.03, .01	.06	.003	.004	.82	.42	-.004, .01
Total effect (c)	-.15	-.46	.20	-2.32	**.02**	-.84, -.07	.12	.14	.08	1.88	.06	-.01, .29
Direct effect (c’)	-.14	-.41	.21	-2.02	**.04**	-.82, -.01	.11	.12	.08	1.58	.12	-.03, .28
	**β**	**B**	**BootSE**	**Boot 95%CI**		**Standardized Boot 95%CI**	**β**	**B**	**BootSE**	**Boot 95%CI**		**Standardized Boot 95%CI**
Indirect effect (a*b)	-.01	-.04	.07	-.18	.09	-.06, .03	.02	.02	.03	-.03	.07	-.03, .06
**Model summary**	**R**^**2**^	**MSE**	**F**	**p**			**R**^**2**^	**MSE**	**F**	**p**		
Outcome	.03	20.51	2.04	.09			.04	2.96	2.41	**.05**		
Total effect model	.03	20.47	2.54	.06			.04	2.95	2.99	**.03**		

*PL* Physical literacy, *MVPA* Moderate-to-vigorous physical activity (minutes per day). The bolded values are statistically significant at a level of *p* < .05

In addition, results indicated that PL was also a significant predictor of total difficulties, B = -0.46, SE = 0.20, 95%CI [-0.84, -0.07], β = -0.15, *p* = 0.02. When MVPA was included in the model, PL was still negatively associated with total difficulties, B = -0.41, SE = 0.21, 95%CI [-0.82, -0.01], β = -0.14, *p* = 0.04, suggesting an increase of one standard deviation in PL was associated with a decrease of 0.14 standard deviations in total difficulties. Nevertheless, no significant mediating role of MVPA was observed between PL and total difficulties, B = -0.04, β = -0.01, Bootstrap 95%CI [-0.06, 0.03]. PL accounts for 10% and 3% of the variance of internalizing problems and total difficulties, respectively.

## Discussion

This cross-sectional study presents movement behaviors, PL, and mental health among children in one community of Canada’s PL4C initiative at baseline. Our sample exhibited a higher level of physical activity than a sample of Canadian children aged 5–9, with 97.3% meeting the PAG and an average of 111.1 min of MVPA per day as measured by accelerometers. According to the most recent surveillance data from the Canadian Health Measures Survey (CHMS), 51.1% of children aged 5–9 years in Canada achieve the recommended PAG, with an average of 68.0 min of MVPA per day [61]. This difference was not entirely surprising. A systematic review evaluating the distinct trajectories of physical activity in children found that physical activity levels decline from 7.7 years old based on objective data [62], suggesting our sample were likely at their most physically active age. In addition, our sample spent comparable hours (2.1 h/day) on recreational screen use with children aged 6–11 (2.1–2.3 h/day) [63]. Our sample obtained an average of 9.8 h/day and 90.4% of them met the SG, which were consistent with the average sleep duration (10 h/day) and SG adherence (86.8%) reported for Canadian children aged 5–9 by the CHMS [61]. In addition, consistent with previous studies [64–67], children in our study accumulated more MVPA and less screen time during school days than weekends. Moreover, we found that these differences existed regardless of whether boys or girls met or did not meet the 24-H movement guidelines. Overall, our baseline device-measured data is consistent with trends in the literature with some indication of the sample being more active than comparable population data.

Our sample showed a similar level of overall motor competence and self-perceived PL to previous studies using PLAY tools with similar age groups [32, 68, 69]. Boys and girls were categorized as "emerging" on the PLAYfun total score and on each domain individually. Children are expected to demonstrate "competent” proficiency in most fundamental movement skills (16/18) by grade 4 [70]. Boys were more proficient in upper body and lower body object control than girls, but less proficient in locomotor skills. However, no gender difference on the PLAYfun total score was observed. This is partially in line with previous findings that Canadian boys outperformed girls on the PLAYfun total score as well as on the domains of running, upper body, and lower body object control [45]. Similar differences in two object control domains were reported in the construct validation study of PLAYfun [46]. Our sample also demonstrated the highest competence in lower body object control skills, in contrast to previous literature that suggested the lowest competence was observed in this domain [45, 47]. However, we found the lowest competence in locomotor skills, which was consistent with Cairney et al. [46].

A mean score of approximately 69 for self-perceived PL measured using the PLAYself tool sets a benchmark for tracking over the next two time points. Consistent with previous studies [32, 45], no significant differences between boys and girls were found. However, other research has observed higher scores in the self-description domain of PLAYself among boys compared to girls when a wider age range [8–14] or youth were examined [70, 71]. Previous studies suggest that maturation affects children’s physical self-perceptions differentially by gender [72]. Additionally, the relationship between the timing of biological maturation and physical activity varies among boys and girls, potentially mediated by many biological and psychological factors including self-esteem [73]. Therefore, it is reasonable to assume that confidence in performing physical activity might be impacted by the gender maturation process each child goes through. Notably, our results indicated that the correlation between PLAYfun and PLAYself was significant, but weak (*r* = 0.15). In addition, PLAYfun displayed a weak to moderate positive association (*r* = 0.30) with MVPA which was double the association between PLAYself and MVPA (*r* = 0.15). Physical competence together with self-perceived competence have been shown to influence the maintenance of children’s physical activity participation [74]. It will be informative to monitor the change in the relationship between physical competence and perceived PL, and how their associations with MVPA change among children over time.

Previous longitudinal studies suggest a wide range of decreases (2.2—38 min/weekday/year and 3.1—41 min/weekend/year) in MVPA with age from childhood to adolescence [75, 76]. For example, overall physical activity was reported to decline by 4.2% per year after the age of 5 [77], and 7.0% per year after the age of 10 [78]. With a baseline sample size of 355 children, it offers us the potential to examine MVPA change and the aforementioned relationships over a two-year period. Required sample size was estimated based on effect sizes (0.28 – 0.45) of MVPA change over time in Nadar et al. [75]. G*Power ver. 3 [79] was used with α = 0.05, 80% power for paired t tests, resulting in an estimated 199 participants needed for a conservative modest effect size (0.2) at time 2 allowing for a 20% dropout rate from baseline and a 70% wear time compliance rate of wrist-worn accelerometers at the second time point [49].

In addition, we found no gender difference in overall PL, which was represented by a PL composite score. In alignment with our study, Caldwell and colleagues also reported that there was no difference in PL between boys and girls [32]. Other research suggested that children who met the PAG display a higher PL [31], which was not evaluated in our study as a high proportion (97.3%) of our sample were physically active. However, we found that PL composite score was still positively associated with MVPA (*r* = 0.27) in this physically active sample. This implies that PL may have a continuous effect on increasing physical activity, or vice versa, among children who meet the current PAG.

This is the first study exploring whether MVPA mediates the associations between PL assessed by PLAY tools and mental health among children aged 7–9 years. A similar previous study using the CAPL reported that PL was positively associated with all aspects of children’s psychosocial well-being assessed by the SDQ, with no mediating role of MVPA [40]. In contrast, our study indicated that PL was not associated with externalizing problems. In addition, when MVPA was included in the model, PL was no longer correlated with prosocial behavior when gender and age were controlled for. The current study demonstrated that MVPA may have a weak but significant direct impact on internalizing problems, but not other aspects of psychosocial well-being or total psychological difficulties. The significant inverse relationship between MVPA and internalizing problems was not found by Melby et al., (2022) [40], but this was in line with other previous studies [80, 81]. Notably, Page et al. (2010) found only significant associations between MVPA and internalizing problems when sedentary behavior was controlled for [81].

Consistent with Melby et al. (2022) [40], we observed a direct effect of PL on total psychological difficulties, but neither a direct nor a mediating effect of MVPA. Similarly, this finding was consistent with Caldwell et al. (2020) who suggested that the direct relationship between composite PL score and health-related quality of life was not mediated by MVPA [32]. A possible explanation could be that children’s PL, as operationalized by movement skills and perceived competence, is related to their overall mental health irrespective of their MVPA. This is understandable as our results support the view that MVPA was linked to internalizing and externalizing problems in different directions [40, 81, 82], although the latter path was not significant in the current study. Alternatively, PL plays a greater role in psychological well-being of children aged 7–9 since they are generally very active at school entry age. Interestingly, our results indicated a small but significant indirect effect of PL on children’s internalizing problems through the effect of MVPA. It is worth noting that the internalizing problems of Melby et al.’s sample were even greater than those of our sample, although both scores were low (3.36 versus 2.9) and the difference was marginal. The discrepancies between the findings of our study and Melby et al.’s study may be attributable to the measurements of PL and MVPA, and children’s demographic factors. While CAPL and PLAY tools were both designed to assess children's PL, their primary focus is different, with CAPL also assessing domains (e.g., physical fitness and physical activity behavior) that can be measured objectively, and other domains (i.e., “motivation and confidence” and “knowledge and understanding”) whereas PLAY tools focus on physical competence [83]. Melby et al.’s study included Danish children with a wider age range from 7 to 13 years, and measured MVPA using thigh-taped accelerometers. In general, the mediation results need to be interpreted with caution, as this study used cross-sectional data, which has limited power for determining causal relationships. The longitudinal nature of the WAVES study will allow for ongoing assessment of the inter-relationships between these variables over time.

Limitations of the present baseline study should be noted. First, our sample was recruited from one school district in West Vancouver. This is an area with higher average household income, higher levels of education, lower unemployment rates, and lower vulnerability in children’s early childhood compared to either metro Vancouver or the British Columbia province [84]. These factors limit the generalizability of our findings to children living in less affluent communities. Second, children’s PLAYbasic skills were not assessed by the same raters who assessed the remaining PLAYfun skills. Third, participants wore accelerometers on their dominant wrists to increase compliance. Wrist-placed accelerometers estimate comparable but consistently higher levels of different intensities of physical activity than waist-worn accelerometers [50, 85]. This may explain to some extent the higher level of MVPA observed in our study. Fourth, we followed the method that Caldwell et al. (2018) used to generate a composite score of PL as each PLAY tool only reflects some domains of PL [32]. However, the PLAYself tool itself does not directly assess knowledge or motivation.

## Conclusions

At the baseline of this two-year longitudinal project embedded within the PL4C initiative, most children were meeting physical activity guidelines, and all children were at an ‘emerging’ level of motor competence. In the first study using PLAY tools in assessing the relationships between PL and children’s mental health, our exploratory results suggest that PL has an independent association with children’s internalizing problems and total difficulties, and MVPA can partially mediate the former relationship. Future cycles of data collection will allow for investigating 1) the trajectories of PL (PLAYfun and PLAYself) with age and as a function of PL implementation support within schools; 2) the inter-relationships between these measure of PL, MVPA and mental wellbeing; and 3) the impact of other factors (e.g., physical and social environmental factors) on the relationship between PL and physical activity.

## Supplementary Information

**Additional file 1: Supplementary 1. **Differences in movement behaviors during school days and weekends by gender and the achievement of Canadian 24-H movement guidelines.

**Additional file 2: Supplementary 2. **Comparisons in movement behaviors between school days and weekends stratified by gender and the achievement of Canadian 24-H movement guidelines.

## Data Availability

The datasets used and/or analyzed during the current study are available from the corresponding author GF on reasonable request.

## Abbreviations

ACTS-MG: Activity Support Scale for Multiple Groups
CAPL: Canadian Assessment of Physical Literacy
CHMS: Canadian Health Measures Survey
CI: Confidence interval
LPA: Light physical activity
MPA: Moderate physical activity
MVPA: Moderate-to-vigorous physical activity
NEWS-Y: Neighborhood Environment Walkability Scale for Youth
PAG: Physical activity guidelines
PL: Physical literacy
PLAY: Physical literacy Assessment for Youth
PL4C: Physical Literacy for Communities
SBG: Sedentary behavior guidelines
SDQ: Strengths and Difficulties Questionnaire
SG: Sleep guidelines
VPA: Vigorous physical activity
