# Supplementary material for: Physical Literacy for Communities (PL4C): physical literacy, physical activity and associations with wellbeing
Source: BMC Public Health. 2023 Jun 29;23:1266. doi: 10.1186/s12889-023-16050-7 (PMC10311742; doi:10.1186/s12889-023-16050-7)
Supplement: Supplementary file 1 — Additional file 1: Supplementary 1. Differences in movement behaviors during school days and weekends by gender and the achievement of Canadian 24-H movement guidelines. [file 12889_2023_16050_MOESM1_ESM.docx]

**Supplementary 1.**

*Differences in movement behaviors during school days and weekends by gender and the achievement of Canadian 24H movement guidelines*

|  | Gender | | | | | Meeting 24-H movement behavior guidelines | | |
| --- | --- | --- | --- | --- | --- | --- | --- | --- |
|  | Overall | Boys | Girls | Statistical test^a^ | Other | Yes | No | Statistical test^a^ |
| **N** | 340 | 172 | 162 |  | 6 | 108 | 142 |  |
| **School days** |  |  |  |  |  |  |  |  |
| Screen time (h/d) | 1.6 (1.2) | 1.8 (1.3) | 1.4 (1.1) | ***U* = 11043.00, *p* < .001** | 2.3 (1.6) | 0.8 (0.5) | 2.3 (1.3) | ***U* = 1519.50, *p* < .001** |
| Sedentary (min/d) | 450.4 (57.9) | 451.1 (58.6) | 449.7 (57.6) | *t*(252) = 0.18, *p* = .85 | 449.1 (58.3) | 434.7 (56.4) | 460.7 (57.0) | ***t*(248) = 3.59, *p* < .001** |
| Sedentary (%) | 56.1 (6.2) | 55.9 (5.7) | 56.4 (6.7) | *t*(252) = -0.72, *p* = .47 | 52.6 (6.3) | 54.7 (6.0) | 57.1 (6.3) | ***t*(248) = 3.03, *p* = .003** |
| MVPA (min/d) | 115.5 (30.9) | 117.7 (30.3) | 113.1 (31.8) | *U* = 7160.50, *p* = .12 | 118.4 (12.7) | 118.8 (30.5) | 113.2 (31.7) | *U* = 7108.50, *p* = .32 |
| MVPA (%) | 14.4 (3.6) | 14.6 (3.5) | 14.1 (3.8) | *U* = 7288.00, *p* = .19 | 13.9 (1.2) | 14.9 (3.5) | 14.0 (3.7) | *U* = 6767.00, *p* = .11 |
| Wear Time (min/d) | 803.4 (59.3) | 807.0 (62.8) | 798.3 (55.0) | *t*(252) = 1.78, *p* = .08 | 853.5 (56.2) | 795.7 (60.0) | 807.7 (59.1) | *t*(248) = 1.59, *p* = .11 |
| **Weekend** |  |  |  |  |  |  |  |  |
| Screen time (h/d) | 2.7 (1.5) | 2.8 (1.6) | 2.5 (1.5) | ***U* = 12157.00, *p* = .04** | 3.1 (1.2) | 1.6 (0.7) | 3.5 (1.5) | ***U* = 1591.50, *p* < .001** |
| Sedentary (min/d) | 476.6 (88.9) | 487.1 (91.6) | 465.7 (85.9) | *t*(252) = 1.92, *p* = .06 | 482.3 (70.8) | 456.1 (78.3) | 492.4 (94.7) | ***t*(248) = 3.23, *p* < .001** |
| Sedentary (%) | 60.1 (8.8) | 60.6 (9.1) | 59.5 (8.5) | *t*(252) = 1.00, *p* = .32 | 60.0 (9.1) | 58.8 (8.1) | 61.3 (9.2) | ***t*(248) = 2.25, *p* = .03** |
| MVPA (min/d) | 100.8 (41.1) | 101.5 (45.4) | 99.9 (36.7) | *U* = 7822.50, *p* = .68 | 106.8 (42.6) | 100.8 (38.0) | 100.4 (43.4) | *U* = 7498.00, *p* = .76 |
| MVPA (%) | 12.7 (5.0) | 12.6 (5.4) | 12.7 (4.5) | *U* = 7594.00, *p* = .42 | 13.3 (5.4) | 12.9 (4.7) | 12.5 (5.2) | *U* = 7204.00, *p* = .41 |
| Wear Time(min/d) | 793.2 (88.3) | 802.7 (87.8) | 783.0 (89.1) | *t*(252) = 1.17, *p* = .24 | 806.6 (45.4) | 777.4 (87.3) | 803.0 (88.3) | ***t*(248) = 2.28, *p* = .02** |

*Note.* Min/d = minutes per day, h/d = hours per day, MVPA = Moderate-to-vigorous physical activity

^a^Independent t-test was used for parametric variables; Mann-Whitney U test was used for non-parametric variables.
