# Supplementary material for: Physical Literacy for Communities (PL4C): physical literacy, physical activity and associations with wellbeing
Source: BMC Public Health. 2023 Jun 29;23:1266. doi: 10.1186/s12889-023-16050-7 (PMC10311742; doi:10.1186/s12889-023-16050-7)
Supplement: Supplementary file 2 — Additional file 2: Supplementary 2. Comparisons in movement behaviors between school days and weekends stratified by gender and the achievement of Canadian 24-H movement guidelines. [file 12889_2023_16050_MOESM2_ESM.docx]

**Supplementary 2.**

Comparisons in movement behaviors between school days and weekends stratified by gender and the achievement of Canadian 24-H movement guidelines

|  |  | | Boys | |  |  | Girls | |  |
| --- | --- | --- | --- | --- | --- | --- | --- | --- | --- |
|  | N | School days | | weekends | Statistical test^a^ | N | School days | weekends | Statistical test^a^ |
| Screen time (h/d) | 172 | 1.8 (1.3) | | 2.8 (1.6) | **Χ^2^ (1, N = 172) = 103.36, *p* < .001** | 162 | 1.4 (1.1) | 2.5 (1.5) | **Χ^2^ (1, *N*= 162) = 113.06, *p*< .001** |
| Sedentary (min/d) | 128 | 451.1 (58.6) | | 487.1 (91.6) | ***F*(1, 127) = 21.65, *p* < .001** | 126 | 449.7 (57.6) | 465.7 (85.9) | ***F*(1, 125) = 5.61, *p* = .02** |
| Sedentary (%) | 128 | 55.9 (5.7) | | 60.6 (9.1) | ***F*(1, 127) = 45.80, *p* < .001** | 126 | 56.4 (6.7) | 59.5 (8.5) | ***F*(1, 125) = 22.33, *p* < .001** |
| MVPA (min/d) | 128 | 117.7 (30.3) | | 101.5 (45.4) | **Χ^2^ (1, *N*= 128) = 34.03, *p*< .001** | 126 | 113.1 (31.8) | 99.9 (36.7) | **Χ^2^ (1, *N*= 126) = 4.57, *p =* .03** |
| MVPA (%) | 128 | 14.6 (3.5) | | 12.6 (5.4) | **Χ^2^ (1, *N*= 128) = 33.27, *p*< .001** | 126 | 14.1 (3.8) | 12.7 (4.5) | **Χ^2^ (1, *N*= 126) = 6.73, *p = .*01** |
| Wear Time (min/d) | 128 | 807.0 (62.8) | | 802.7 (87.8) | *F*(1, 127) = 0.33, *p* = .57 | 126 | 798.3 (55.0) | 783.0 (89.1) | ***F*(1, 125) = 4.34, *p* = .04** |
|  |  |  | | Meeting 24-H movement guidelines | | | | |  |
|  |  | Yes | | |  |  | No | |  |
| Screen time (h/d) | 108 | 0.8 (0.5) | | 1.6 (0.7) | **Χ^2^ (1, *N*= 108) = 74.71, *p*< .001** | 142 | 2.3 (1.3) | 3.5 (1.5) | **Χ^2^ (1, *N*= 142) = 79.07, *p*< .001** |
| Sedentary (min/d) | 108 | 434.7 (56.4) | | 456.1 (78.3) | ***F*(1, 107) = 10.88, *p* = .001** | 142 | 460.7 (57.0) | 492.4 (94.7) | ***F*(1, 141) = 16.44, *p* < .001** |
| Sedentary (%) | 108 | 54.7 (6.0) | | 58.8 (8.1) | ***F*(1, 107) = 35.88, *p* < .001** | 142 | 57.1 (6.3) | 61.3 (9.2) | ***F*(1, 141) = 36.74, *p* < .001** |
| MVPA (min/d) | 108 | 118.8 (30.5) | | 100.8 (38.0) | **Χ^2^ (1, *N*= 108) = 17.93, *p*< .001** | 142 | 113.2 (31.7) | 100.4 (43.4) | **Χ^2^ (1, *N*= 142) = 14.90, *p*< .001** |
| MVPA (%) | 108 | 14.9 (3.5) | | 12.9 (4.7) | **Χ^2^ (1, *N*= 108) = 19.59, *p*< .001** | 142 | 14.0 (3.7) | 12.5 (5.2) | **Χ^2^ (1, *N*= 142) = 16.46, *p*< .001** |
| Wear Time (min/d) | 108 | 795.7 (60.0) | | 777.4 (87.3) | ***F*(1, 107) = 7.50, *p* = .01** | 142 | 807.7 (59.1) | 803.0 (88.3) | *F*(1, 141) = 0.37, *p* = .55 |

*Note.* Min/d = minutes per day, h/d = hours per day, MVPA = Moderate-to-vigorous physical activity

^a^One-way repeated measures ANOVA was used for parametric variables; Friedman’s test was used for non-parametric variables.
